# Sex-dependent effects of carbohydrate source and quantity on caspase-1 activity in the mouse central nervous system

**DOI:** 10.1186/s12974-024-03140-5

**Published:** 2024-06-05

**Authors:** Rasa Valiauga, Sarah Talley, Mark Khemmani, Melline Fontes Noronha, Rocco Gogliotti, Alan J. Wolfe, Edward Campbell

**Affiliations:** 1 Stritch School of Medicine, Loyola University Chicago, Maywood, IL USA; 2 Department of Microbiology and Immunology, Loyola University Chicago, Maywood, IL USA; 3 Department of Molecular Pharmacology and Neuroscience, Loyola University Chicago, Maywood, IL 60153 USA; 4 Edward Hines Jr. VA Hospital, Hines, IL 60141 USA

**Keywords:** High glycemic index diet, Ketogenic diet, Glucose intolerance, Inflammation, Inflammasome, Neuroinflammation, Caspase-1, Sex, Microbiome, Behavior

## Abstract

**Background:**

Mounting evidence links glucose intolerance and diabetes as aspects of metabolic dysregulation that are associated with an increased risk of developing dementia. Inflammation and inflammasome activation have emerged as a potential link between these disparate pathologies. As diet is a key factor in both the development of metabolic disorders and inflammation, we hypothesize that long term changes in dietary factors can influence nervous system function by regulating inflammasome activity and that this phenotype would be sex-dependent, as sex hormones are known to regulate metabolism and immune processes.

**Methods:**

5-week-old male and female transgenic mice expressing a caspase-1 bioluminescent reporter underwent cranial window surgeries and were fed control (65% complex carbohydrates, 15% fat), high glycemic index (65% carbohydrates from sucrose, 15% fat), or ketogenic (1% complex carbohydrates, 79% fat) diet from 6 to 26 weeks of age. Glucose regulation was assessed with a glucose tolerance test following a 4-h morning fast. Bioluminescence in the brain was quantified using IVIS in vivo imaging. Blood cytokine levels were measured using cytokine bead array. 16S ribosomal RNA gene amplicon sequencing of mouse feces was performed to assess alterations in the gut microbiome. Behavior associated with these dietary changes was also evaluated.

**Results:**

The ketogenic diet caused weight gain and glucose intolerance in both male and female mice. In male mice, the high glycemic diet led to increased caspase-1 biosensor activation over the course of the study, while in females the ketogenic diet drove an increase in biosensor activation compared to their respective controls. These changes correlated with an increase in inflammatory cytokines present in the serum of test mice and the emergence of anxiety-like behavior. The microbiome composition differed significantly between diets; however no significant link between diet, glucose tolerance, or caspase-1 signal was established.

**Conclusions:**

Our findings suggest that diet composition, specifically the source and quantity of carbohydrates, has sex-specific effects on inflammasome activation in the central nervous system and behavior. This phenotype manifested as increased anxiety in male mice, and future studies are needed to determine if this phenotype is linked to alterations in microbiome composition.

**Supplementary Information:**

The online version contains supplementary material available at 10.1186/s12974-024-03140-5.

## Background

An estimated 55 million people worldwide suffer from dementia, with the prevalence expected to drastically increase in the coming years as the population ages [1]. The term dementia describes a range of cognitive disorders characterized by progressively declining memory, executive function, and ability to carry out activities of daily living due to the underlying neuronal degeneration. The most common form of senile dementia is Alzheimer’s disease, and a combination of genetic, environmental, and lifestyle factors likely contribute to the pathophysiology [2–4]. Dementia is one of the leading causes of morbidity and mortality worldwide, affecting not only individual and caregiver quality of life but the economy, with financial cost associated with dementia constituting about 1% of the global gross product [5]. As current treatments are largely ineffective, elucidating the roles of potential players in the pathogenesis and progression of this debilitating disease can lead to novel therapeutic targets.

Mounting evidence suggests metabolic dysregulation may be a key factor in the onset and progression of neurodegenerative disorders, with type 2 diabetes mellitus conferring a nearly two-fold greater risk of developing dementia [6–11]. The primary characteristic of diabetes mellitus is hyperglycemia, which is the driver of micro- and macro-vascular complications of diabetes such as retinopathy, neuropathy, and cerebrovascular disease. Glucose intolerance is the inability to regulate blood sugar in the body effectively and over time, persistent glucose intolerance can contribute to the development of hyperglycemia and diabetes. Sustained elevation in blood glucose also contributes to chronic low-grade inflammation, as glucose induces oxidative stress, activates immune cells, promotes transcription of proinflammatory genes, and increases proinflammatory cytokines in the blood [12–14].

Inflammation is a hallmark feature of both metabolic and neurodegenerative diseases, and in recent years the inflammasome has emerged as a potential link between these pathologies [15, 16]. Inflammasomes are cytosolic multiprotein complexes that activate innate inflammatory responses, primarily through promoting the maturation and release of proinflammatory cytokines [17]. Although they play a critical role in host defense against infections, inflammasome dysregulation has been implicated in numerous pathologies, including metabolic and neurodegenerative diseases [18–20]. While several inflammasomes with unique structures and functions have been identified (reviewed in [21–25]), they all share some common features. The key components of an inflammasome are (1) a sensor protein that recognizes pathogen-associated molecular patterns (PAMPs) and damage-associated molecular patterns (DAMPs), (2) an adaptor protein containing a caspase-recruitment domain, and (3) the effector protein, which is an inflammatory caspase (caspase-1, -4, -5, or -11).

The most widely studied inflammasome is the nucleotide-binding oligomerization domain family pyrin domain containing 3 (NLRP3) inflammasome. The NLRP3 inflammasome is composed of three proteins: NLRP3, apoptosis-associated speck-like protein containing a caspase activating recruitment domain (ASC), and caspase-1. Canonical NLRP3 inflammasome activation is a two-step process, with priming triggered by pattern recognition receptors (PRRs) at the transcriptional/translational level and assembly of the proteins activated by PAMPs and DAMPs. Upon assembly, active caspase-1 cleaves pro-IL-1β, pro-IL-18, and gasdermin-D (GSDMD) into their active forms, leading to the release of proinflammatory interleukins IL-1β and IL-18 and induction of pyroptosis by GSDMD. Diet plays a significant role in regulating the activity of the inflammasome, with several dietary factors identified as potential modulators [26–30].

Diet is also known to influence the development and progression of metabolic disorders such as diabetes, and there is literature to suggest that dietary modifications are beneficial in dementia [31, 32]. Diets with a high glycemic index, which contain carbohydrates that are quickly metabolized and cause a rapid increase in blood sugar, have been shown to cause inflammation and behavioral deficits in rodents [33–35]. In contrast, studies examining low carbohydrate, high fat ketogenic diets have demonstrated improved cognitive function in mice, in part through modulating neuroinflammatory processes [36–39].

However, few animal studies examining the connection between diet and neuroinflammatory outcomes have incorporated sex as a consideration, despite knowledge that metabolism, immune responses, and risk of dementia are highly influenced by sex hormones [40–42]. Many researchers choose to exclusively use male mice to avoid the potential physiological variability associated with the estrous cycle, although recent findings suggest that the estrous cycle does not influence female mouse behavior [43]. Additionally, investigators prefer to use male mice in metabolic studies since males are more responsive to dietary interventions [44–46]. This sex bias has led to a limited understanding of the female brain and poor translation to human studies, which is why our study incorporates both male and female mice.

Here we used a high glycemic index diet and a ketogenic diet to establish whether the source and quantity of carbohydrates in the diet can influence systemic inflammation and CNS inflammasome activation, and determine if sex specific sensitivities exist for any of these variables. We found that the ketogenic diet caused metabolic derangements in both male and female mice, but that the high glycemic index diet in males and the ketogenic diet in females led to increased central nervous system inflammasome activation. These findings correlated with elevated IL-1β and TNF levels in the serum and the emergence of anxiety-like phenotypes. We observed that although high glycemic index diet and the ketogenic diet induced significant changes in the fecal microbiomes, these changes were not significantly linked with changes in caspase-1 activity or glucose tolerance.

## Results

### Ketogenic diet leads to weight gain and glucose intolerance in male and female mice

At 6 weeks of age, mice were started on a control (CD), high glycemic (HGD), or ketogenic (KD) diet, which was maintained until 26 weeks. The HGD is identical to the CD in macronutrient composition but all the carbohydrates in the HGD are derived from sucrose. Conversely, the KD is nearly devoid of carbohydrates and is composed primarily of plant-based fats (Additional file 1).

**Fig. 1:**
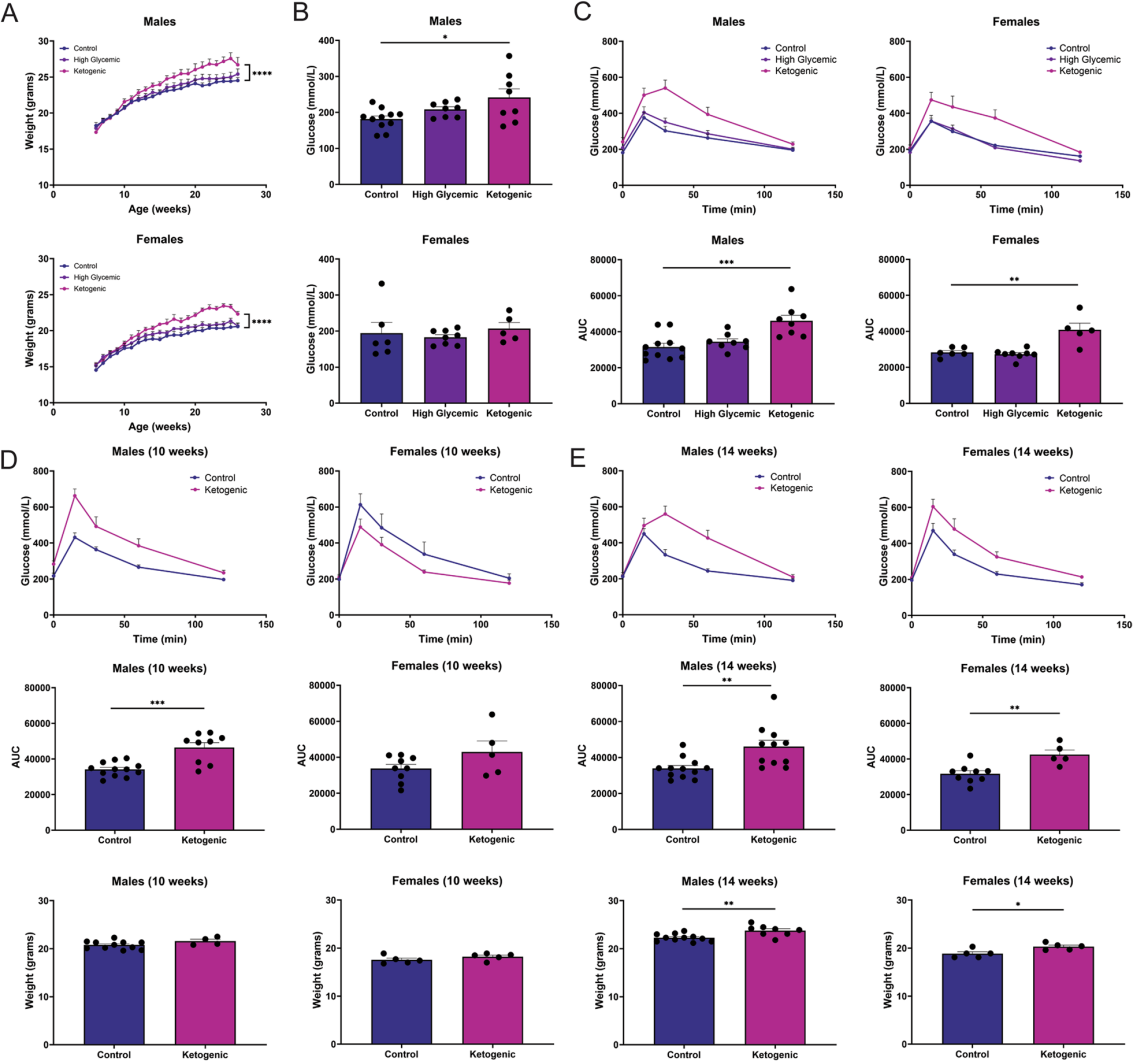
Ketogenic diet leads to weight gain and glucose intolerance in male and female mice. **A** Body weight was measured prior to diet intervention and weekly thereafter until the end of the experiment, at 26 weeks. Difference in body weight was assessed using a mixed effects model for high glycemic index (HGD) and ketogenic (KD) diets relative to control diet (CD). **B** Fasting glucose was measured following a 4-h morning fast at 26 weeks. **C** Mice were injected with a glucose solution and blood glucose was measured at 15-, 30-, 60-, and 120-min post injection. Area under the curve (AUC) for the glucose tolerance test (GTT) was calculated as an index of whole glucose excursion. **D** Male mice on the KD first displayed impaired glucose tolerance at 10 weeks, which did not correspond to weight gain, while **E** female mice on the KD first showed impaired glucose at 14 weeks, coinciding with significant weight gain. All data are shown as mean ± SEM and n = 5–11 per group. Statistical differences for weight over time were calculated with a mixed effects model. One-way ANOVA followed a Dunnett’s test was used to compare multiple groups (**A–C**) and a Student’s *t* test was used to compare two groups (**D**, **E**). For all statistical tests, *, **, ***, ****p < 0.05, 0.01, 0.001, and 0.0001, respectively

To assess the metabolic effects of each diet, we measured body weight weekly and tested glucose tolerance monthly. To confirm the KD induced ketosis, we performed a β-hydroxybutyrate assay to assess the level of ketone bodies and found significant elevations of this ketone in both male and female mice at 26 weeks (Additional file 2). Male and female mice on the KD gained significantly more weight relative to mice on the CD (Fig. 1A). Fasting blood glucose is a simple and common method of assessing glucose regulation and is often used as a screening tool for prediabetes and diabetes in the clinic. In rodents, the most common assessment of glucose homeostasis is the glucose tolerance test (GTT), which evaluates the ability of the body to move sugar from the blood into tissues. Abnormal glucose tolerance generally precedes impaired fasting glucose. At 26 weeks, male mice fed the KD had higher fasting blood glucose relative to sex matched controls, while female fasting glucose was unaffected (Fig. 1B). Between the two test diets, a GTT revealed that only the KD led to impaired glucose homeostasis, which was observed in both male and female mice (Fig. 1C).

Impaired glucose tolerance was first observed at 10 weeks in males (Fig. 1D) and at 14 weeks in females (Fig. 1E), 4 weeks and 8 weeks after diet initiation, respectively. Glucose intolerance is often associated with obesity, but we found that the intolerance in males preceded any substantial weight gain, which was not statistically significant until 14 weeks and remained modest at that time (Fig. 1D). In females, glucose intolerance corresponded to a mild but significant increased body weight at 14 weeks (Fig. 1E). Together, these results suggest that the KD, high in plant-based fat and nearly devoid of carbohydrates, causes metabolic dysregulation in male and female mice with a C57BL/6 background.

### Diet composition influences caspase-1 activation in the brains of mice in a sex-dependent manner

To evaluate how changes in diet influence inflammasome activation in the central nervous system (CNS), we utilized mice expressing a caspase-1 biosensor that allows caspase-1 activation to be monitored in vivo [47–50]. These mice express a circularly permuted luciferase that is inactive prior to activation of caspase-1. The cleavage of caspase-1 causes the two terminal ends of luciferase to come together and generates an enzymatically active luciferase molecule, allowing caspase-1 activation to be monitored in vivo and ex vivo [47]. To directly monitor biosensor activation in the CNS in vivo, a modified cranial window technique was performed on 5-week-old mice, wherein the skull was thinned, and a layer of transparent cyanoacrylate glue was applied to protect the cranium [51].

**Fig. 2:**
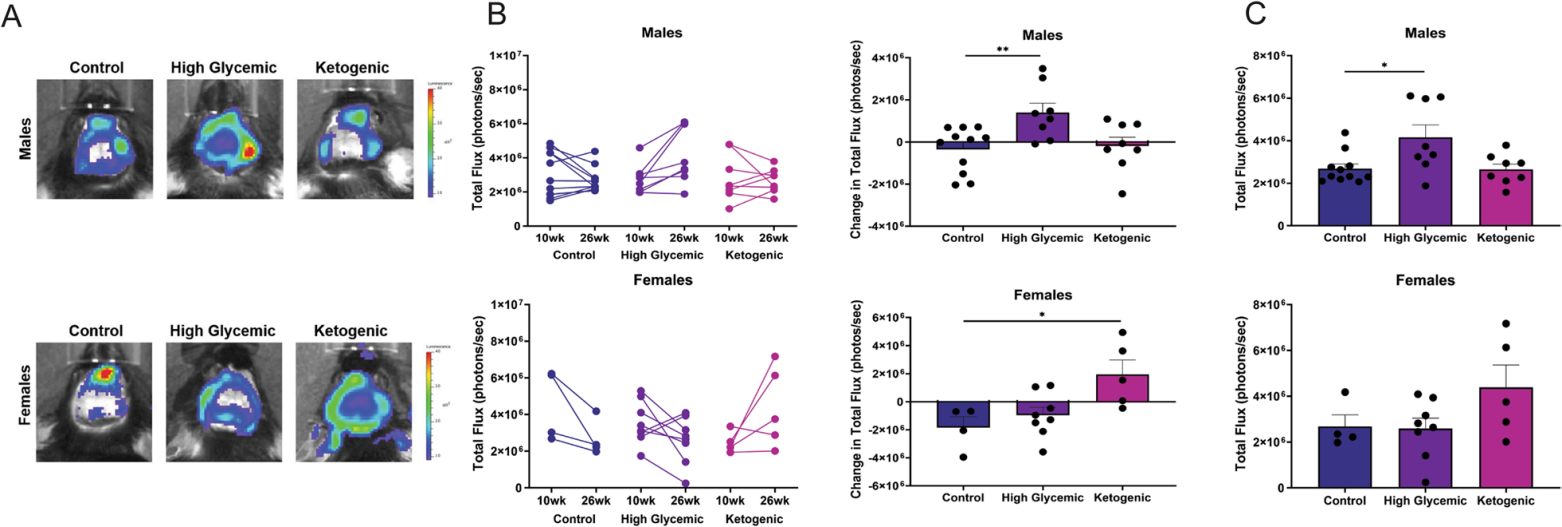
High glycemic index diet (HGD) in males and ketogenic diet (KD) in females causes increased caspase-1 activation in the brain. **A** Representative images of caspase-1 biosensor mice with cranial windows at 26-weeks. **B** Cleaved caspase-1 signal was measured in mice at 10- and 26-weeks of age and the difference between the two time points was calculated for each animal. **C** Caspase-1 signal at 26 weeks in males and females. All data are shown as mean ± SEM and n = 4–11 per group. Statistical differences were determined using one-way ANOVA followed by a Dunnett’s test, comparing the HGD or KD to their sex-matched CD, where *, **p < 0.05 and 0.01 respectively

We observe that this procedure induces transient caspase-1 activation at the procedure site, which resolves in 3 to 5 weeks (Additional file 3). We therefore recorded biosensor activation at 10 weeks, when biosensor activation induced by the surgery had subsided, and at 26 weeks of age. We found that the HGD in males and the KD in females caused a significant increase in caspase-1 activation over the course of the study when week 10 and week 26 time points for individual animals were compared (Fig. 2A, B). At 26 weeks, male mice fed the HGD displayed significantly more caspase-1 activation than males on the CD (Fig. 2C, top panel), consistent with the changes observed over time, while a trend towards an increase was observed in female mice receiving the KD, although this difference was not statistically significant (Fig. 2C, bottom panel). A two-way ANOVA revelated that there was a significant interaction between the effects of diet and sex on caspase-1 activation (F(2,38) = 9.046, p = 0.0006). These data suggest that inflammasome activity in the brain is sensitive to diet changes and the carbohydrate load and type affects males and females differently.

### IL-1β and TNF in the serum correlate with caspase-1 activation in the CNS

**Fig. 3:**
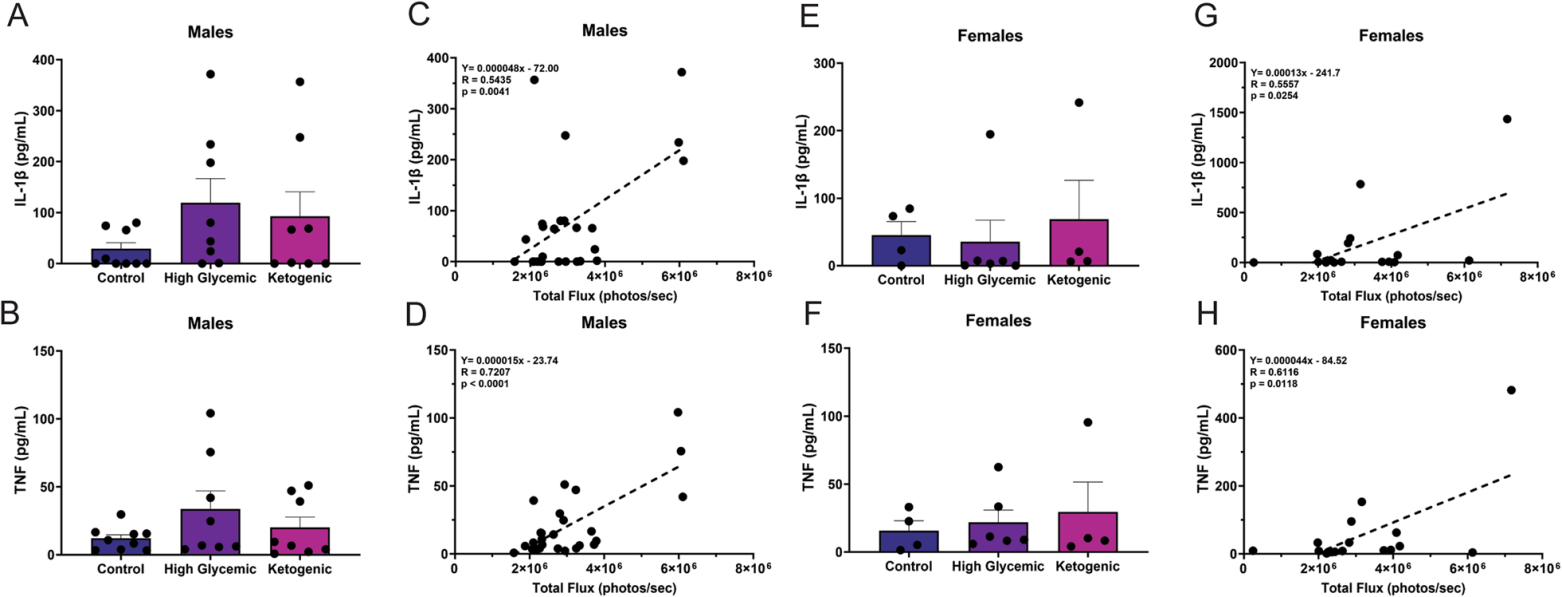
IL-1β and TNF in the serum correlate with caspase-1 activation in the brain. Cytokine levels in serum collected from 26-week-old mice were quantified using a CBA. **A** IL1-β and **B** TNF levels were measured in the serum of male mice. Correlation between **C** IL-1β and **D** TNF in the serum and caspase-1 activation in the CNS was assessed using simple linear regression in males. **E** IL-1β and **F** TNF levels were measured in the serum of female mice. Correlation between **G** IL-1β and **H** TNF in the serum and caspase-1 activation in the CNS was assessed using simple linear regression in females. All data are shown as mean ± SEM and n = 4–10 per group. Statistical differences were determined using one-way ANOVA followed by a Dunnett’s test, comparing the HGD or KD to their sex-matched CD. Correlation analysis of serum cytokines and caspase-1 activation were performed using a simple linear regression

To define the systemic changes in inflammation induced by HGD and KD, we quantified the presence of proinflammatory cytokines and chemokines in the serum collected from mice at 26 weeks using a cytokine bead array (CBA). Only IL-1α in male mice fed the KD was significantly elevated (Additional file 4). However, we did note that, although statistically insignificant, IL-1β and TNF levels showed a pattern consistent with biosensor activation that was elevated with the HGD in males (Fig. 3A, B) and the KD in females (Fig. 3E, F). We assessed the correlation of these cytokines with the brain caspase-1 activation and found a significant association between IL-1β (R = 0.5435, P = 0.0041 for males; R = 0.5557, P = 0.0254 for females) and TNF (R = 0.7207, P < 0.0001 for males; R = 0.6116, P = 0.0118 for females) in the serum and biosensor signal in the brain in both males (Fig. 3C, D) and females (Fig. 3G, H). These data reveal that significant increases in CNS inflammation may not be reflected in significant changes in serum cytokines, as measured by CBA. However, the strong correlation between biosensor activation in the CNS and serum cytokine levels further demonstrate that the differences in biosensor activation due to dietary changes underlie differences in diet-driven inflammatory responses.

### Diet composition influences the fecal microbiome

Given the substantial body of evidence suggesting the gut microbiome regulates glucose metabolism (reviewed in [52]) and modulates neuroinflammation (reviewed in [53]), we performed 16S ribosomal RNA gene sequencing of mouse feces collected at 26 weeks on the same day the bioluminescence brain measurements were taken and serum was collected for cytokine analysis. We focused on the male microbiome given the statistically significant increase in CNS caspase-1 activation observed in these animals at 26 weeks. A total of 26 samples (10 CD, 8 HGD, 8 KD) were analyzed; 1 CD sample (sample 4) was excluded from further analysis because it differed substantially from all other samples (Additional file 5), and thus skewed downstream analyses.

Raw measurements of area under the curve for the glucose tolerance test and total flux for caspase-1 activation were each binned into either normal or high (at least one standard deviation outside of the mean of CD measurement). Our analyses revealed no significant link between diet and glucose tolerance (Additional file 6), or diet and caspase-1 signal (Additional file 7). However, we did observe significant differences in the composition of the mouse fecal microbiome between diets.

**Fig. 4:**
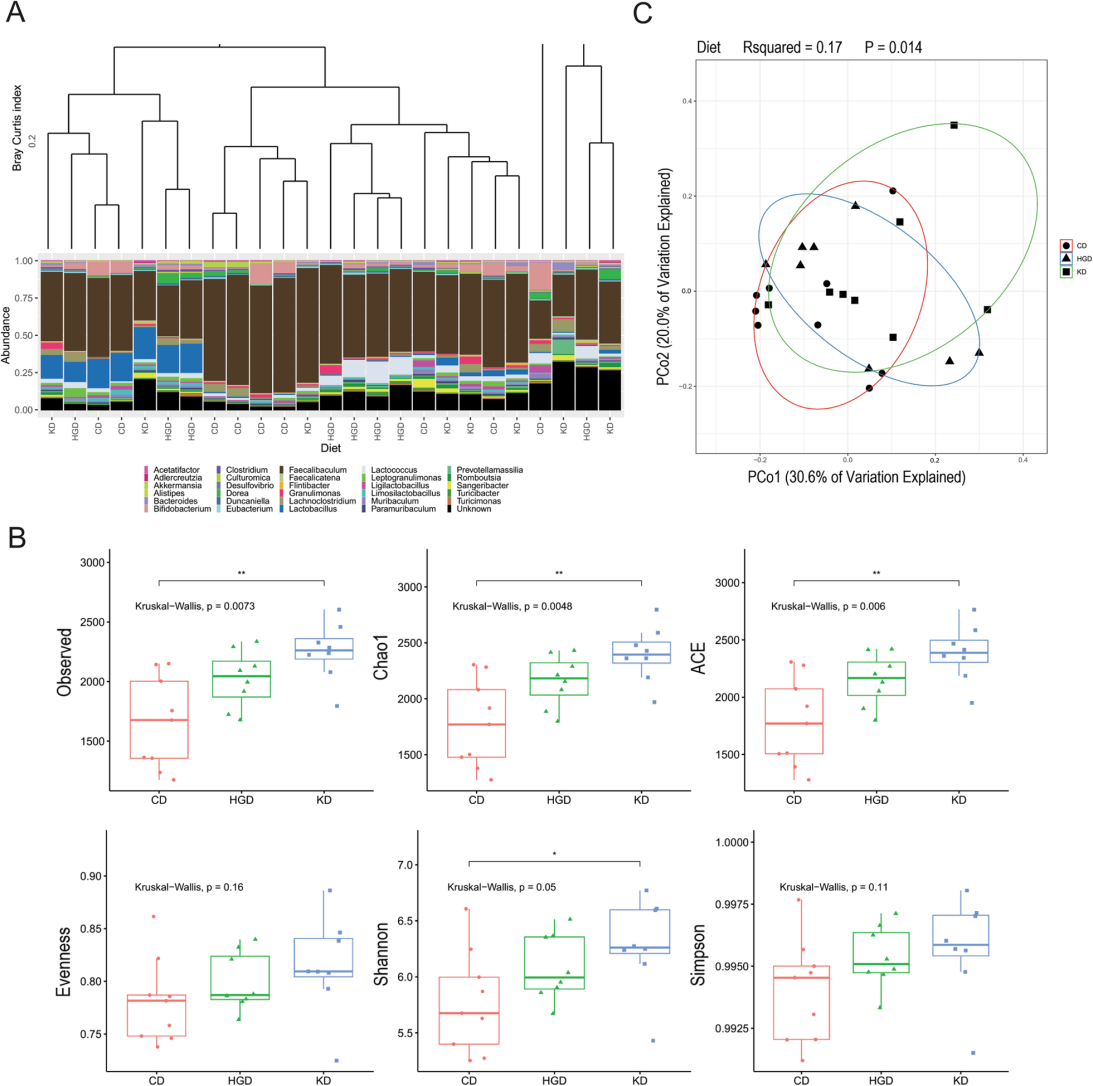
Diet alters the male mouse microbiome. DNA was extracted from 26-week-old male mice and 16S ribosomal RNA gene amplicon sequencing performed. **A** Bray–Curtis Dissimilarity Index identified clusters of samples that were similar in composition. **B** Alpha diversity analysis was performed by calculating richness (ace, chao1, and observed richness), evenness (Pielou) and overall diversity (Shannon and Simpson). **C** Beta diversity was assessed by PCoA using the Bray–Curtis Dissimilarity Index at the ASV level and resultant values tested for significance with the experimental covariates using the PERMANOVA test. N = 8–10 per group. Statistical comparisons of bacterial abundance were conducted using the Phyloseq package and involved pairwise Chi-Squared Tests, and results were filtered based on a significance threshold of Benjamini-Hochberg adjusted p-value < 0.01

**Fig. 5:**
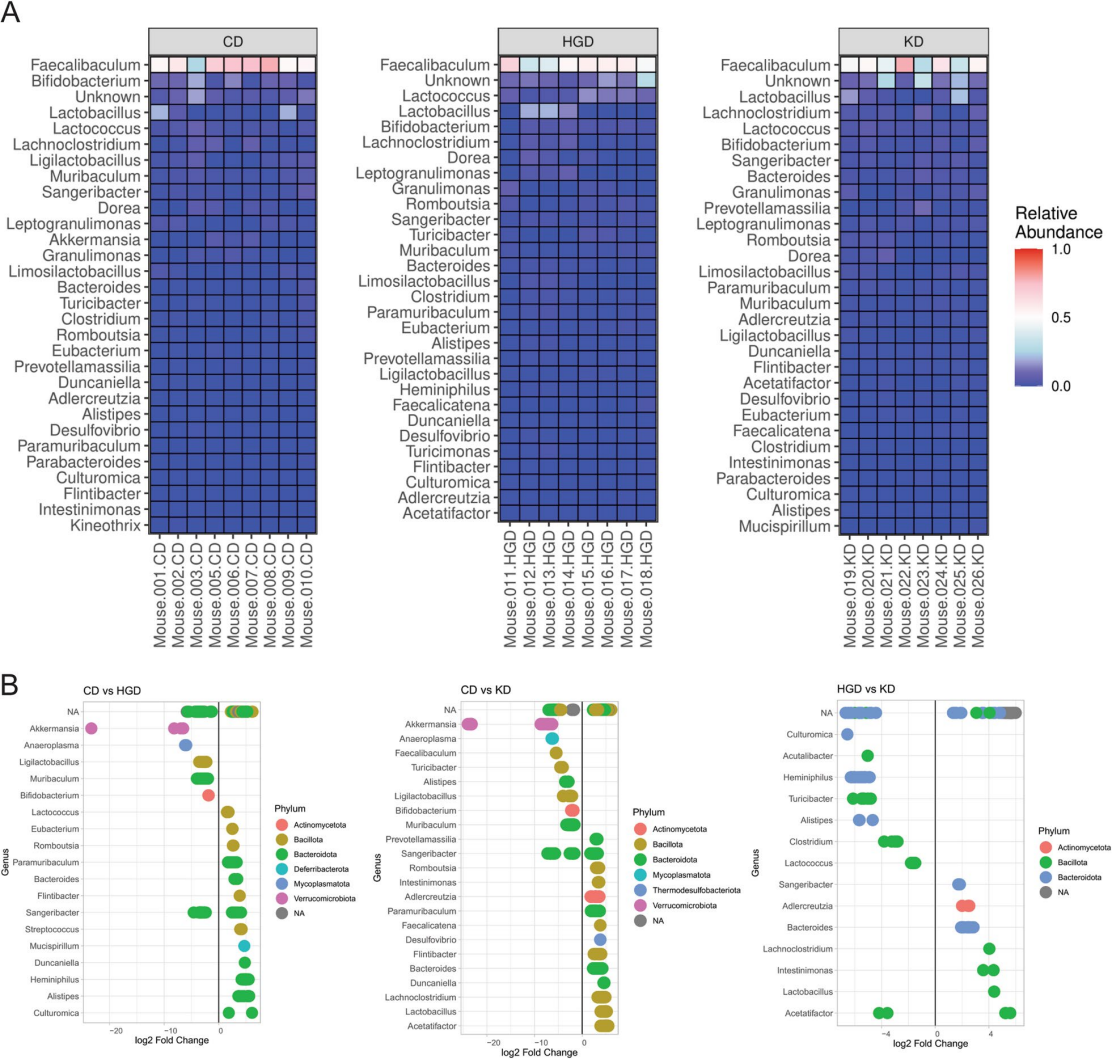
Specific genera of bacteria are enriched in the high glycemic index and ketogenic diets. DNA was extracted from 26-week-old male mice and 16S ribosomal RNA gene amplicon sequencing performed. **A** The top 30 genera displayed as a relative abundance heatmap. **B** DESeq analysis of bacterial abundance between diets, where individual dots represent different amplicon sequence variants within the genus. N = 8–10 per group. Statistical comparisons of bacterial abundance were conducted using the DESeq package and involved pairwise Chi-Squared Tests, and results were filtered based on a significance threshold of Benjamini-Hochberg adjusted p-value < 0.01

The Bray–Curtis Index identified clusters of samples that were similar in composition. Whereas the genus *Faecalibaculum* predominated in all fecal samples, the subgroups clustered as a function of their non-*Faecalibaculum* composition (Fig. 4A). These clusters generally related to diet. For example, in mice fed the KD relative to mice on the CD, alpha (within sample) diversity analysis revealed a significant increase in 3 different measures (Observed, Chao1, and ACE) of richness (number of unique taxa) and 1 measure of overall diversity (Shannon) (Fig. 4B). Beta diversity (between sample) analysis revealed significant differences in microbial composition between diets (Fig. 4C). These differences resulted primarily from the relative abundance of the genera *Lactobacillus*, *Bifidobacterium*, and *Lactococcus* (Fig. 5A). In the CD samples, the greatest relative abundance was *Faecalibaculum*, followed by *Bifidobacterium* and *Lactobacillus*. In the HGD samples, the greatest relative abundance was *Faecalibaculum*, followed by *Lactococcus* and *Lactobacillus*. In the KD samples, the greatest abundance was once again *Faecalibaculum*, followed by *Lactobacillus*. Finally, we used DEseq analysis to more deeply compare compositions and identify several additional genera significantly enriched in the experimental diets compared to each other and to the control (Fig. 5B). Together, these data indicate that diet composition significantly alters the microbiome but that changes in glucose intolerance and caspase-1 activation in the brain are not associated with specific changes in the fecal microbiome.

### Carbohydrate source and quantity alters mouse anxiety behavior, but does not impact motor function or learning and memory

We next sought to evaluate whether the changes in neuroinflammation have an impact on mouse behavior using accelerated rotarod (motor coordination and learning), zero maze (anxiety), novel object recognition (spatial memory), and fear conditioning (associative memory) at 26 weeks. The tests were executed from least invasive (zero maze) to most invasive (fear conditioning) and only one test was performed per day.

**Fig. 6:**
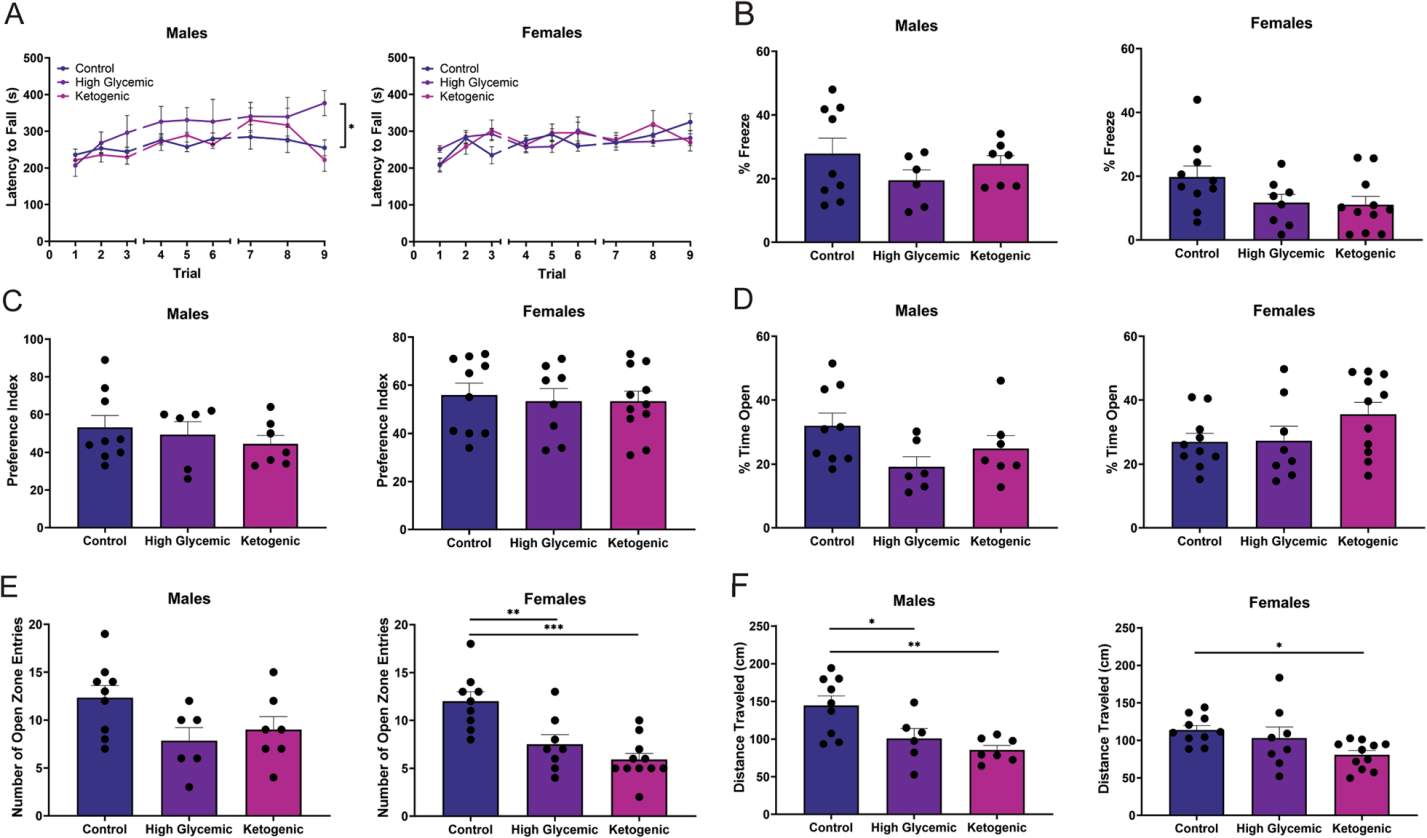
Carbohydrate source and quantity alters mouse anxiety behavior without impacting motor function or learning and memory. C57BL/6 mice were randomly assigned to control (CD), high glycemic index (HGD), or ketogenic (KD) diet at 6 weeks of age and put through a battery of behavior tests at 26 weeks. **A** Accelerated rotarod was used to evaluate motor coordination and learning by recording latency to fall from the rod. **B** Percent freezing in cued fear conditioning was used as a measure of associative memory. **C** Novel object recognition assessed spatial learning. Preference index was defined Time_novel_/(Time_novel_ + Time_familiar_). Elevated zero maze evaluated anxiety-like behavior via **D** percent time spent in open zone, **E** number of open zone entries, and **F** total distance traveled. All data are shown as mean ± SEM and n = 6–11 per group. One-way ANOVA followed by a Dunnett’s test was used to assess statistical significance, comparing the HGD or KD to their sex-matched CD, wherein *, **, ***p < 0.05, 0.01, and 0.001, respectively

In the rotarod test, we observed no meaningful differences between the diets in both males and females (Fig. 6A), suggesting that our experimental paradigm did not alter gross motor function or coordination. Additionally, we assessed if diet composition impacts memory using novel object recognition and fear conditioning tests. We found no differences in spatial learning via novel object recognition or in associative memory via cued fear conditioning, independent of sex or diet (Fig. 6B, C).

Since neuroinflammation is linked to changes in anxiety behaviors, we tested mice in an elevated zero maze (EZM), which incorporates open space and elevation as anxiogenic factors [54, 55]. We found that male mice on the HGD have an increased anxiety-like phenotype, manifesting as significantly decreased total distance traveled (Fig. 6F). Females on the same diet also showed increased anxiety-like behaviors in the form of significantly less entries made to the open zones (Fig. 6E). Neither sex had significant deficits in the time spent in open zones of the EZM on the KD (Fig. 6D). Females on the KD made significantly fewer entries into the open zones (Fig. 6E), which correlated with significantly decreased distance explored (Fig. 6F). A two-way ANOVA revealed that there was not a statistically significant interaction between the effects of diet and sex on number of entries made into the open zones (F = (2,36) = 0.9493, p = 0.3965) or distance traveled (F = (2,45) = 1.5711, p = 0.2191). Together, our phenotypic analysis points to a potential diet-driven link between inflammasome activation and the emergence of anxiety like phenotypes.

## Discussion

Metabolic dysfunction linked to poor dietary choices is increasingly recognized as a contributor to cognitive decline [56–59]. While Mediterranean-style diets have repeatedly been found to improve overall health and prevent cognitive decline, the Western diet has been linked to increased risk of dementia and Alzheimer’s disease [31, 60–63]. The Western diet is high in fat and refined carbohydrates, and there has been research addressing both aspects. Numerous studies have investigated the impact of high fat diet on cognition, with mixed results [64–74]. However, fewer animal studies have focused on high glycemic index diets, composed of refined food that causes rapid spikes in blood glucose, despite clinical findings suggesting that this type of diet causes metabolic impairments, cognitive decline, and increased amyloid load [75–79].

In finding dietary interventions that may counteract neurodegenerative processes, several human studies have assessed the impact of the ketogenic diet on cognitive health [80–83]. Ketogenic diets are high fat, low carbohydrate diets that have shown promising outcomes in patients suffering from refractory epilepsy and promote quick weight loss, which is why they are being explored as a treatment for dementia. However, the clinical trials thus far are small and inconclusive, necessitating the need for further animal studies.

In the current study, we chose to investigate how the effects of high glycemic index and ketogenic diets on glucose regulation influence the neuroinflammatory response. As this pathology is important in disorders like dementia, we posit that linking diet to CNS inflammasome activation would be a salient finding. Importantly, we included both male and female mice because there is a sex bias in dementia (females are more affected) and metabolism of macronutrients is different in males and females. The mice were fed a control (CD), high glycemic index (HGD), or ketogenic (KD) diet from 6 to 26 weeks of age, with continuous monitoring of weight and glucose tolerance.

We found that both male and female mice on the KD gained more weight (Fig. 1A). Many studies have shown that mice fed a KD maintain or lose weight, but most of these investigations are short in duration or restrict food intake [84–87]. In contrast, our study involved 20 weeks of ad libitum feeding and our results are similar to the findings of Goldberg et al., which indicate that while short-term KD feeding improves metabolic parameters, long-term continuous KD feeding results in weight gain, glucose intolerance, and immune dysfunction [88]. We also noted that at 26 weeks, male mice on the KD had higher fasting blood glucose (Fig. 1B). However, only the KD led to impaired glucose tolerance in both sexes (Fig. 1C), which preceded significant weight gain in males at 10 weeks and coincided with weight gain in females at 14 weeks (Fig. 1D, E). These findings are similar to those noted in a recent study using male mice, where the authors observed a ketogenic diet caused glucose intolerance [89]. The delayed glucose intolerance in females is not surprising, as female mice are known to display improved glucose tolerance and increased insulin sensitivity, in part due to the action of sex hormone regulation of metabolism [90–92]. These results suggest that the high-fat, low-carbohydrate composition of the KD causes glucose metabolism dysregulation independent of weight gain in males and in the context of excess body weight in females.

To assess how changes in glucose homeostasis impact neuroinflammation, our study utilized mice expressing a caspase-1 biosensor to monitor inflammasome activation in the CNS [49]. We employed a modified cranial window technique on 5-week-old mice to allow visualization of biosensor activation in the brain [51]. When we compared bioluminescence at 10 and 26 weeks, the data revealed significantly increased caspase-1 activation in males on the HGD and females on the KD over the course of the study (Fig. 2A). The observations associated with the KD differ from a recent investigation where the authors showed the ketogenic diet with a similar proportion of macronutrients as ours reduced neuroinflammation and improved cognitive function [36]. However, their study used 7-month-old 5xFAD male mice, which have a much different metabolic profile and CNS environment. Our data suggests that carbohydrate source and quantity affect inflammasome activity in the brain differently in non-diseased males and females.

The sex-specific differences in CNS caspase-1 activation in response to diet is a novel finding, but there is research to suggest that the inflammasome is regulated differently in male and female mice. For example, Posillico et al. showed that central administration of the immunostimulant polyinosinic:polycytidylic acid induces greater elevation in hippocampal cytokine and chemokine levels in male compared to female mice [93]. In contrast, Chen et al. found that the NLRP3 inflammasome exerts a more significant influence on high fat diet-induced atherosclerosis in female than male mice [94]. This suggests sex-dependent variations in the inflammasome’s response to diverse inflammatory stimuli, largely due to the regulatory effects of sex hormones [95–98]. Considering that inflammasome signaling proteins caspase-1, ASC, and IL-1β are increased in the cortex of aged female mice compared to aged male mice [99], further studies would benefit from a longer timeline or the use of ovariectomized female mice to test whether our phenotype is estrogen-dependent.

We hypothesized that increased inflammasome activation in the CNS may be a result of systemic inflammation, as peripheral inflammation has been shown by our group and others to modulate inflammasome activity in the brain [48, 100–102]. Consistent with this, we found that there was significant correlation between IL-1β and TNF levels in the serum and biosensor signal in the brain in both males and females (Fig. 3C and 3D). Notably, we did not observe a statistically significant increase in these serum cytokines induced by HGD or KD in either males or females, but observed clear, statistically significant changes in biosensor activation over time in animals, as well as a significant correlation between serum cytokines and inflammasome activation in the CNS, as measured by biosensor activation. This underscores the limitations of using systemic cytokines as a measure for tracking minor changes in inflammation caused by diet or other stimuli. It also highlights that alterations in tissue caspase-1 activation can reveal substantial and biologically relevant changes that go beyond what can be observed solely by monitoring serum cytokine levels.

Given substantial evidence that the gut microbiome affects brain health, we performed 16S ribosomal RNA gene amplicon sequencing on the stool isolated from the 26-week-old male mice used in the metabolism and inflammation experiments to explore if the changes in metabolism and neuroinflammation were due to alterations in the gut microbiota. Although the fecal microbiome did not fully explain the glucose intolerance and increased caspase-1 signal in the brain (Fig. S4 and S5), we found significant differences in the composition of microbes of the experimental diets compared to control and each other (Figs. 4, 5), an outcome that has been previously documented in studies using other diet paradigms [103–107].

The link between microbiome composition and inflammasome activity is a complex, bidirectional relationship that has garnered significant attention in recent years in the pathophysiology of numerous diseases. Several studies have demonstrated that certain bacteria can influence inflammasome activation and downstream inflammatory processes by production of metabolites that are sensed by the inflammasome (reviewed in [108–111]). For example, acetate is a short-chain fatty acid produced by commensal gut microbiota inhibits the NLRP3 inflammasome in a calcium dependent manner [112]. Conversely, lipopolysaccharide produced by gram negative in the gut is a known endotoxin and activator of the inflammasome [113–116]. Therefore, we explored whether the changes in CNS caspase-1 activation and glucose intolerance were byproducts of diet-associated changes in the microbiome. Although we did not find a statistically significant link, this is likely due to our relatively small sample size and stringent statistical cutoffs for significance in the analysis. Nonetheless, it is important to frame the study in the context of the microbiome as it is well known that murine gut microbiota differ significantly between institutions and can affect experimental outcomes [117–121].Lastly, we evaluated whether our test diets influence mouse behavior due to the established link between neuroinflammation, neurodegeneration, and cognitive deficits. We observed no significant differences in males or females with respect to motor coordination and motor learning (Fig. 6A), spatial memory (Fig. 6B), or associative memory (Fig. 6C) at 26 weeks. However, we found that male mice on the HGD had significantly increased anxiety, traveling less distance within the maze (Fig. 6E), compared to male mice fed the CD. Female mice on the KD also showed increased anxiety-like behaviors, with less entries made into the open zones (Fig. 6E) and decreased distance explored (Fig. 6E). These results demonstrate that diets with a high glycemic index or those extremely high in fat influence the anxiety phenotype in rodents in a sex-dependent manner and manifest prior to memory impairment, a mental health issue known to occur in the early stages of Alzheimer’s disease in humans (reviewed in [122, 123]).

One major strength of our investigation is that we investigated two diets that are in direct opposition with respect to the carbohydrate source and quantity within a single study that followed the mice over a period of approximately 5 months in both males and females. To our knowledge, ours is the first study to examine sex-specific differences in diet-induced CNS inflammasome activation. Another strength of our study is the use of a caspase-1 biosensor mouse model, which allowed us to track changes in vivo in the same mouse, permitting for a more direct and accurate comparison of changes and reducing the number of animals needed for research.

However, there are several limitations and technical considerations that necessitate discussion. The main pitfall of our study is the relatively small sample size, which possibly led us to missing important findings. It is also important to consider the effect of the cranial window surgery on the neuroinflammatory response. While we find that the acute induction in caspase-1 activation resolves within a month, it is impossible to know whether the baseline is altered. Whereas this may not be an issue when exploring changes in inflammasome activity with strong insults such as lipopolysaccharide or traumatic brain injury models, this could diminish our ability to detect small differences due to dietary modifications.

Additionally, given that this is a skull-thinning rather than the traditional cranial window procedure, the thickness of the window is potentially not uniform across the skull or identical from mouse to mouse. Given the blinded nature of the surgery (i.e., the future diet assignment was not known at the time of the surgery), it is unlikely that this caveat biased our results, but it is possible that this introduced additional variability. Regarding the utility of our caspase-1 biosensor mouse, we and others have validated this tool in various disease models, including in the CNS [47–51]. However, the sensitivity of in vivo brain imaging has yet to be determined and it is possible that our diets induced meaningful, yet not statistically significant, caspase-1 cleavage. Nonetheless, this system is the most direct way to measure inflammasome activation that is currently available and should be considered in future studies examining inflammation in the CNS.

Lastly, is important to address that age as a major limitation, our investigation only followed mice into adulthood. Dysregulation of inflammasome signaling is implicated in the pathogenesis metabolic dysfunction and dementia, both of which are characterized by chronic inflammation, impaired glucose metabolism, and disrupted insulin signaling. The most significant risk factor for metabolic and neurodegenerative diseases is aging and there is strong evidence to support inflammasomes are critical in driving the chronic low-grade inflammatory state associated with aging, termed “inflammaging” [124–128]. Nonetheless, our findings are important as researchers and clinicians consider using dietary interventions for preventative purposes. Overall, our results suggest that the type and amount of carbohydrates in food can influence neuroinflammation and behavior differently in male and female mice. Future studies are necessary to define the underling mechanisms responsible our findings.

## Conclusions

We observed sex specific differences in inflammatory responses in the CNS to HGD and KD, with a HGD promoting CNS inflammasome activation in males and a KD promoting CNS inflammasome activation in females. This phenotype was associated with behavior alterations, specifically increased anxiety. This emphasizes the importance of sex as a biological variable in dietary studies. Additionally, we found that utilization of a caspase-1 biosensor to monitor inflammasome activation over time in living animals can detect mild inflammatory responses in the CNS induced by dietary changes that cannot be reliably detected by monitoring serum cytokine levels.

## Methods

### Animals and experimental design

Caspase-1 biosensor or C57BL/6 mice were bred in-house and maintained in pathogen free conditions at either Loyola University Chicago or Edward Hines, Jr. VA Hospital. At 5 weeks of age, caspase-1 biosensor mice were fitted with cranial windows. At 6 weeks, mice were randomly assigned to a control, high glycemic index, or ketogenic diet using blocked randomization. For behavioral studies, C57BL/6 mice were randomly assigned to one of the diets at 6 weeks using blocked randomization. Mice were fed ad libitum for the duration of the study. Investigators were not blinded to diet assignment moving forward. All mice were housed in a standard animal facility with a 12-h alternating light/dark cycle. All experiments were performed according to protocols approved by Loyola University Chicago Institutional Animal Care and Use Committee.

### Diet formulations

Three purified rodent diets from Research Diets, Inc. were used and contained the following macronutrients as %kcal: control diet (65% carbohydrates, 20% protein, 15% fat), high glycemic diet (65% carbohydrates, 20% protein, 15% fat), and ketogenic diet (1% carbohydrates, 20% protein, 79% fat). It should be noted that the composition of the control and high glycemic diets is the same regarding percentages from macronutrients, but all the carbohydrates in the high glycemic diet are derived from sucrose. To avoid any confounding results, the control and ketogenic diets are devoid of any sucrose or dextrose. Complete formulations are found in the supplemental material (Additional file 1).

### Cranial window procedure

A thinned-skull cranial window technique was employed to visualize the brain, as described previously [51]. Mice were anesthetized with intraperitoneal injections of tribromoethanol and placed on a heating pad to maintain body temperature during surgery. Once appropriate depth of anesthesia was achieved as determined by reflex testing of the footpad, the mouse head was stabilized on a stereotaxic frame to maintain head position and ophthalmic ointment applied to the eyes. The head was sterilized using 70% ethanol and Betadine. The epidermis and muscle layers were excised to expose the skull by making a sagittal incision through the scalp, cutting as needed to fully expose the skull. The skull was cleaned of any blood and connective tissue attached to the skull was scraped off with a surgical blade. A micro-drill was used to thin the skull, moving quadrant to quadrant and making sure not to thin in any one area for an extended amount of time to avoid friction induced overheating. Saline solution was applied intermittently to dissipate any heat and wash away debris. Once a thickness of ~ 20 µm has been achieved, the skull was thoroughly cleaned and dried, cyanoacrylate glue (PELCO Pro C1000) applied over the entire area, and a few of drops of Insta-Set CA Accelerator (Bob Smith Industries, Inc) were added to rapidly cure the glue. Mice were given long-acting buprenorphine post-op to minimize any discomfort.

### Bioluminescence measurements

Mice were anesthetized with isoflurane, injected intraperitoneally with 150 mg/kg VivoGlo Luciferin and skulls imaged 10 min later using the IVIS 100 Imaging System (Xenogen). Regions of interest were drawn around the tissue, and bioluminescence was quantified within each region using Living Image software.

### Validation of ketogenic diet intervention

Serum was isolated from whole blood collected from the submandibular at 26 weeks. A β-hydroxybutyrate Colorimetric Assay Kit (Cayman Chemical) was used to assess levels of ketone bodies.

### Glucose tolerance test

Following a 4-h morning fast, a 12.5% dextrose solution was injected intraperitoneally at 1 g/kg body weight and blood glucose measurements were taken at baseline, 15-, 30-, 60-, and 120-min post injection. Glucose levels were assessed via a tail blood sample using the AlphaTRAK2 glucometer and appropriate testing strips (Zoetis US, Parsippany-Troy Hills, NJ).

### Cytokine and chemokine measurements

Serum was isolated from whole blood collected from the submandibular. Levels of proinflammatory cytokines and chemokines were measured using cytokine bead array (BD Biosciences), according to the manufacturer’s protocol. Samples were incubated with capture and detection beads for a selected panel of cytokines and chemokines: IL-1α, IL-1β, IL-4, IL-6, TNF, KC, MCP-1, MIP-1α and MIP-1β. Samples were measured on an LSRFortessa (BD Biosciences) and analyzed using FCAP Array software v3.0 (BD Biosciences).

### Microbiome analysis

16S ribosomal RNA gene amplicon sequencing was performed on male mouse feces collected at 6 months, simultaneously with the bioluminescence brain measurements and serum collection for cytokine analysis. We focused on the male microbiome due to the larger sample size and because differences in caspase-1 activation at 26 weeks were only significant in male mice. Genomic DNA was extracted from feces as follows: the fecal pellet was resuspended in 1 mL of sterile PBS and 100 μL of the suspension was transferred to deep-well plates. Enzymatic lysis solution consisting of 4 mg Lysozyme, 3kU Mutanolysin, 4 mg Lysostaphin, and 200U Achromopeptidase in buffer (20 mM Tris, 2 mM EDTA, 1.2% Triton, pH 8.0) was added to each sample and incubated at 37 ˚C for 5 h, shaking intermittently at 1000 rpm (Eppendorf Thermomixer with SmartBlock DWP1000). Samples were then processed using DNeasy 96 Blood & Tissue Kit (Qiagen, Inc) per the manufacturer's instructions. To assess potential DNA contamination, an extraction negative control (no feces) was processed with samples and sequenced. Specifically, the V4 region of the 16S rRNA gene was amplified using the 515f and 806r primers. The sequencing was carried out by the University of Illinois at Chicago Genome Research Core (UIC GRC), where a two-stage PCR amplification protocol was used to generate amplicons containing Illumina sequencing adapters, a sample-specific barcode sequence, and the amplification target. These amplicons were purified and pooled, then sequenced with paired-end 150 base reads using an Illumina MiSeq instrument.

### Basic processing of microbiome data

Raw reads were quality filtered using FASTQC (v0.11.9) and cutadapt tools (Version 4.0), with reads shorter than 100 bp discarded. Following this, paired-end FASTQ reads were processed through the DADA2 pipeline using default parameters. Taxonomic annotation of the resultant Amplicon Sequence Variants (ASVs) was performed by the BLCA tool and the NCBI 16S Microbial database version 2023 [129]. A total of 26 samples (10 CD, 8 HGD, 8 KD) were analyzed; 1 CD sample (sample 4) was excluded from further analysis because it was substantially different from all other samples (Additional file 4).

### Downstream microbiome analyses

The alpha diversity analysis was performed by calculating richness (ace, chao1 and observed richness), diversity (Shannon and Simpson) and evenness (Pielou) indices in R using the phyloseq and vegan packages [130, 131]. The Bray–Curtis Index was used in beta diversity analysis to identify clusters of samples that are similar in composition. Principle coordinate analysis (PCoA) was calculated using the Bray–Curtis Dissimilarity Index at the ASV level. The resulting values were tested for significance with the experimental covariates using the PERMANOVA test. The top 30 genera are shown as a relative abundance heatmap for each sample. Statistical comparisons of bacterial abundance among different Diet covariates were conducted using the Phyloseq and DESeq2 packages [132]. Statistical analyses involved pairwise Chi-Squared Tests, and results were filtered based on a significance threshold of Benjamini-Hochberg adjusted p-value < 0.01.

### Rotarod

Accelerated Rotarod was used as a general assessment of neuromuscular function and motor learning. Mice were placed on a rotating rod with increasing speed from 4 to 40 rpm over 600 s and latency to fall was recorded. The test was performed three times per day for three consecutive days, with 1 h between each trial.

### Novel object recognition

6-month-old mice were placed inside a chamber with two identical objects (either a beaker or a T75 culture flask) and allowed to explore the chamber for 10 min. The mice were returned to their home cage for 1 h. Following the rest period mice were returned to the chamber, where one of the objects was replaced by a novel object, and again allowed to explore for 10 min. The familiar and novel object were assigned at random and alternated to control for potential inherent preference. The time spent directly sniffing each object was tracked and scored by Noldus Software. The preference index was defined as Time_novel_/(Time_novel_ + Time_familiar_).

### Cued fear conditioning

Training for cued fear conditioning was performed using a 10% vanilla odor cue and two 1-s, 0.7 mA foot shocks spaced 30 s apart that were preceded by a tone. Associative learning was quantified 24 h later as the percent time spent freezing in a novel chamber containing a 10% vanilla odor cue in response to presentation of the tone. Freezing over the test period was quantified using Video Freeze software.

### Elevated zero maze (EZM)

For EZM, mice were placed on an elevated circular platform, with two closed sections comprising 50% of the apparatus, and two open sections, comprising the remaining 50%. The time spent exploring open or closed regions over a 5-min testing period served as a read out of anxiety behavior. The assay was conducted under full light conditions (~ 400 lx) in the open sections and the time spent exploring was quantified by ANY-maze software.

### Statistical analysis

All data are expressed as mean + SEM. Grubbs’ test with α = 0.05 was used to identify and remove outliers. Mixed-effects model was used to analyze changes in body weight over time. For comparison of two groups, a Student’s *t* test was used. For comparison of three groups, one-way ANOVA followed by post-hoc Dunnett’s test was used. Correlation analysis of serum cytokines and caspase-1 activation were performed using a simple linear regression. Statistical significance was set at *p* < 0.05. Graphs were created and calculations performed using GraphPad Prism 10 software (GraphPad Software, Inc.).

### Supplementary Information

Additional file 1. Diet formulations. Experimental and control diets were purchased from Research Diets, Inc.

Additional file 2. Mice on the Ketogenic Diet Exhibit Ketosis. Serum was isolated from 26-week-old mice and β-hydroxybutyrate levels assessed using a commercially available kit. All data are shown as mean ± SEM and n = 5–9 per group. A Student’s *t* test was used to compare the KD to the sex-matched CD, where *p < 0.05 and ***p < 0.001.

Additional file 3. Cleaved caspase-1 signal was measured in mice at 6-, 8-, 10- and 12-weeks of age. Data are shown as mean ± SEM and n = 49. Statistical differences were determined using Student’s *t* test, comparing individual time points, where ****p < 0.0001.

Additional file 4. Inflammatory cytokine/chemokine levels in serum collected from 26-week-old mice. Mice were started on a control, high glycemic index, or ketogenic diet at 6 weeks of age and serum was isolated at 26 weeks. Cytokine/chemokine were measured by CBA in male and female mice. All data are shown as mean ± SEM and n = 3–10 per group. Statistical differences were determined using one-way ANOVA followed by a Dunnett’s test, comparing the HGD or KD to their sex-matched CD. *p < 0.05.

Additional file 5. Relative abundance clustered by the Bray-Curtis Dissimilarity Index for all samples. DNA was extracted from 26-week-old male mice and 16S ribosomal RNA gene amplicon sequencing performed. The Bray-Curtis Dissimilarity Index identified 1 control diet sample (sample 4) that was substantially different and therefore excluded from further analysis. N = 8–10 per group.

Additional file 6. Microbiome composition does not fully explain glucose intolerance phenotype. DNA was extracted from 26-week-old male mice and 16S ribosomal RNA gene amplicon sequencing performed. Raw measurements of area under the curve for the glucose tolerance test were binned into either normal or high and used as metadata in analyses. **(A)** Alpha diversity analysis was performed by calculating richness (ace, chao1, and observed richness), evenness (Pielou) and overall diversity (Shannon and Simpson). **(B)** Beta diversity was assessed by PCoA using the Bray-Curtis Dissimilarity Index at the ASV level and the resultant values tested for significance with the experimental covariates using the PERMANOVA test. N = 8–10 per group. Statistical comparisons of bacterial relative abundance were conducted using the Phyloseq package and involved pairwise Chi-Squared Tests, and results were filtered based on a significance threshold of Benjamini-Hochberg adjusted p-value < 0.01.

Additional file 7. Microbiome composition does not fully explain caspase-1 activation in the CNS. DNA was extracted from 26-week-old male mice and 16S ribosomal RNA gene amplicon sequencing performed. Raw measurements of total flux for caspase-1 activation were binned into either normal or high and used as metadata in analyses. **(A)** Alpha diversity analysis was performed by calculating richness (ace, chao1, and observed richness), diversity (Shannon and Simpson), and evenness (Pielou). **(B)** Beta diversity was assessed by PCoA using Bray-Curtis dissimilarities at the ASV level and dissimilarity indices were tested for significance with the experimental covariates using the PERMANOVA test. N = 8–10 per group. Statistical comparisons of bacterial abundance were conducted using the Phyloseq package and involved pairwise Chi-Squared Tests, and results were filtered based on a significance threshold of Benjamini-Hochberg adjusted p-value < 0.01.

## Data Availability

Sequencing data that support the findings of this study have been deposited in the National Center for Biotechnology Information (NCBI) Archive with the primary accession code PRJNA1067688.

## Abbreviations

ASC: Apoptosis-associated speck-like protein containing a caspase activating recruitment domain
ASV: Amplicon sequence variant
CD: Control diet
CNS: Central nervous system
DAMP: Damage-associated molecular pattern
EZM: Elevated zero maze
GSDMD: Gasdermin-D
GTT: Glucose tolerance test
HGD: High glycemic index diet
KD: Ketogenic diet
NLRP3: Nucleotide-binding oligomerization domain family pyrin domain containing 3
PAMP: Pathogen-associated molecular pattern
PCoA: Principle coordinate analysis
PRR: Pattern recognition receptors
