# Supplementary figures and images for: Sex-dependent effects of carbohydrate source and quantity on caspase-1 activity in the mouse central nervous system
Source: J Neuroinflammation. 2024 Jun 5;21:151. doi: 10.1186/s12974-024-03140-5 (PMC11155082; doi:10.1186/s12974-024-03140-5)

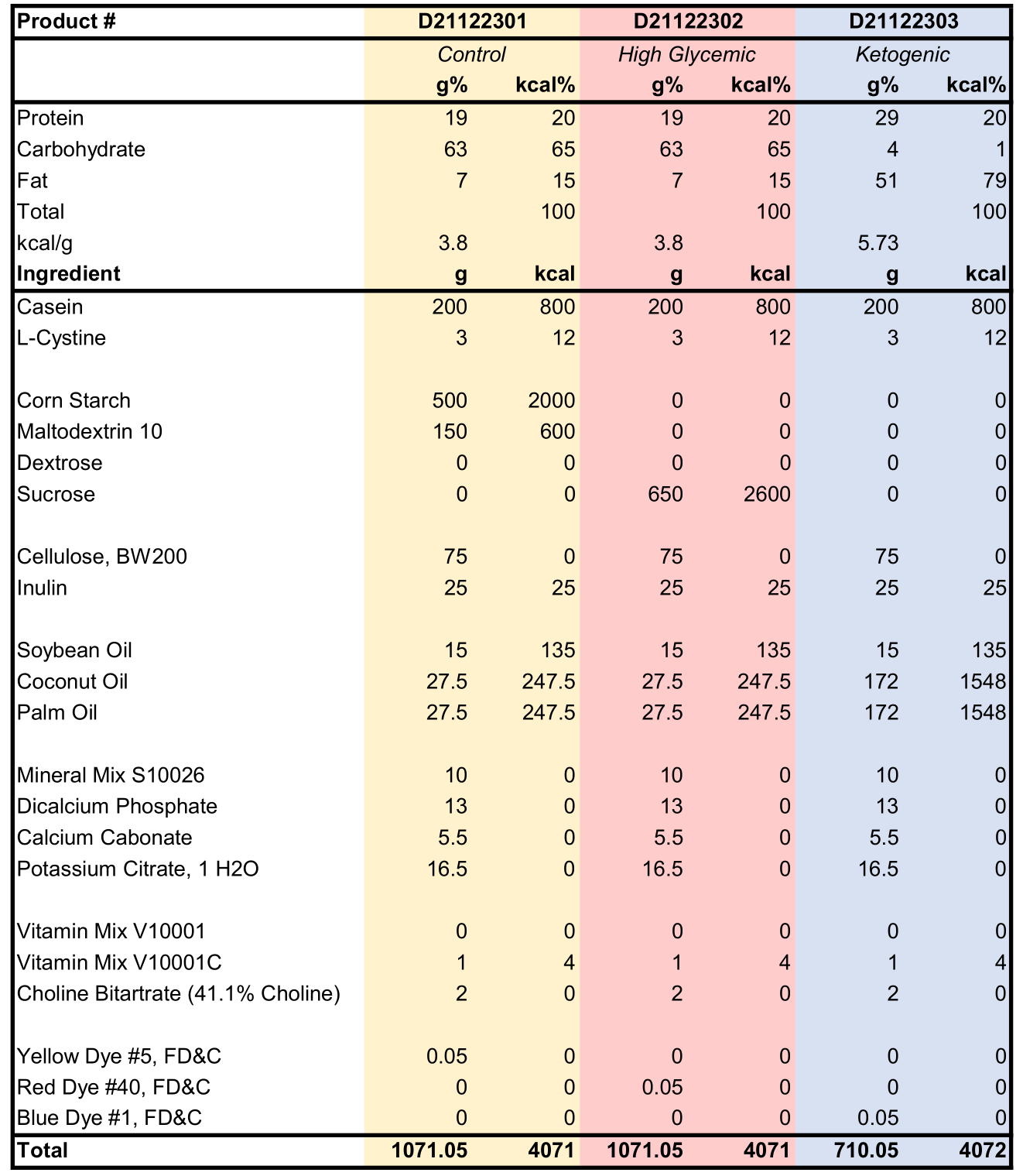

Supplement: Supplementary file 1 — Additional file 1. Diet formulations. Experimental and control diets were purchased from Research Diets, Inc. [file 12974_2024_3140_MOESM1_ESM.tif]

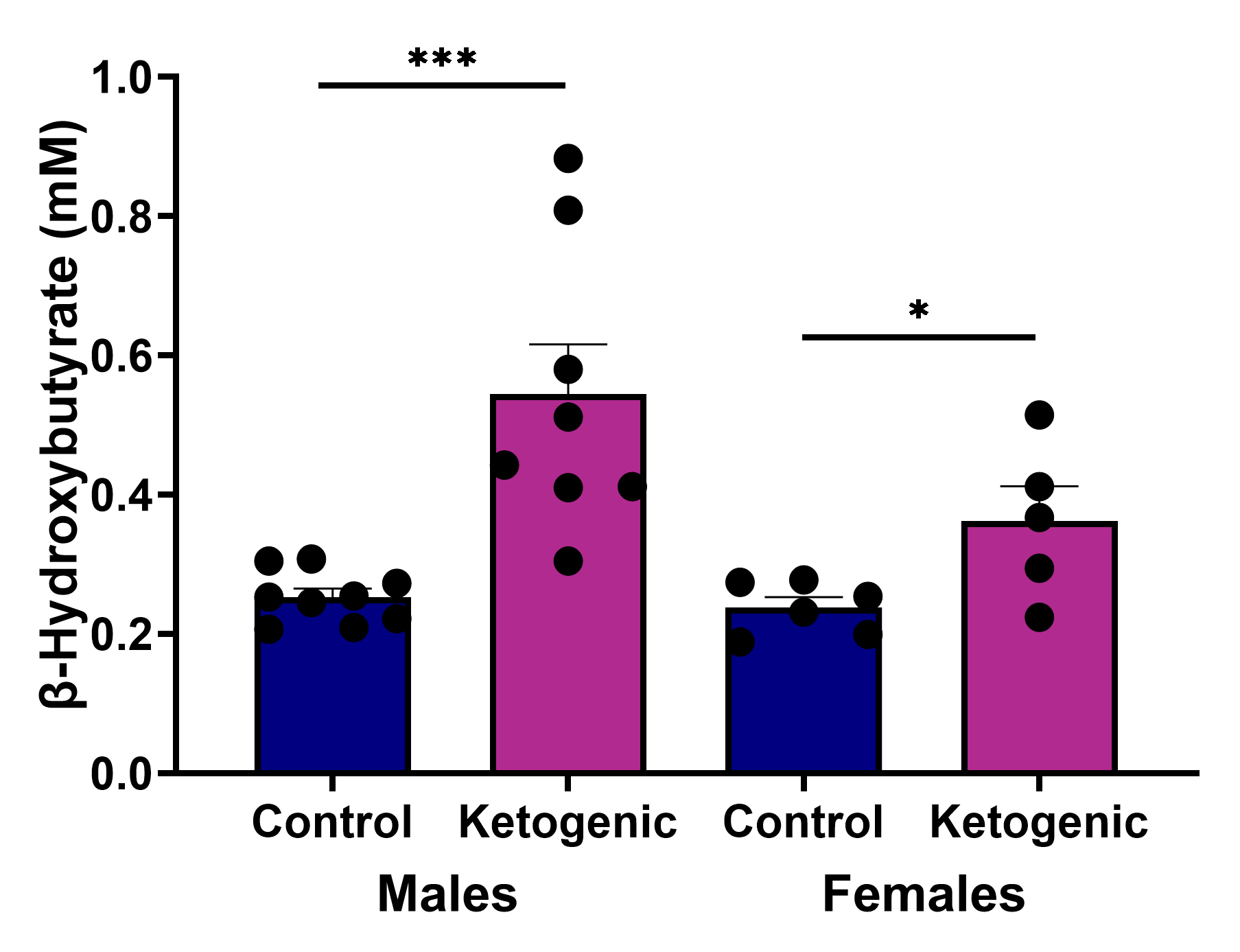

Supplement: Supplementary file 2 — Additional file 2. Mice on the Ketogenic Diet Exhibit Ketosis. Serum was isolated from 26-week-old mice and β-hydroxybutyrate levels assessed using a commercially available kit. All data are shown as mean ± SEM and n = 5–9 per group. A Student’s t test was used to compare the KD to the sex-matched CD, where *p < 0.05 and ***p < 0.001. [file 12974_2024_3140_MOESM2_ESM.tif]

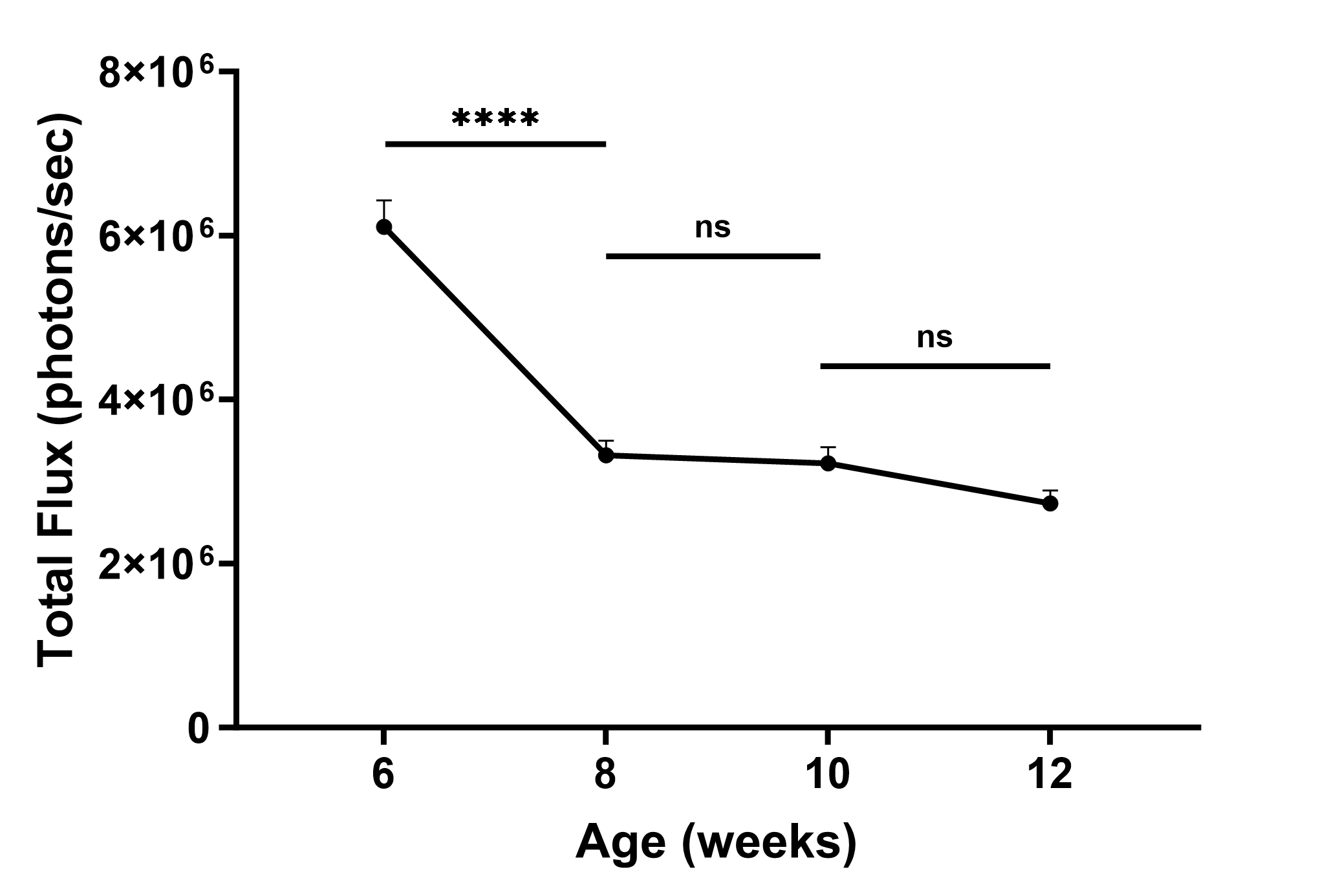

Supplement: Supplementary file 3 — Additional file 3. Cleaved caspase-1 signal was measured in mice at 6-, 8-, 10- and 12-weeks of age. Data are shown as mean ± SEM and n = 49. Statistical differences were determined using Student’s t test, comparing individual time points, where ****p < 0.0001. [file 12974_2024_3140_MOESM3_ESM.tif]

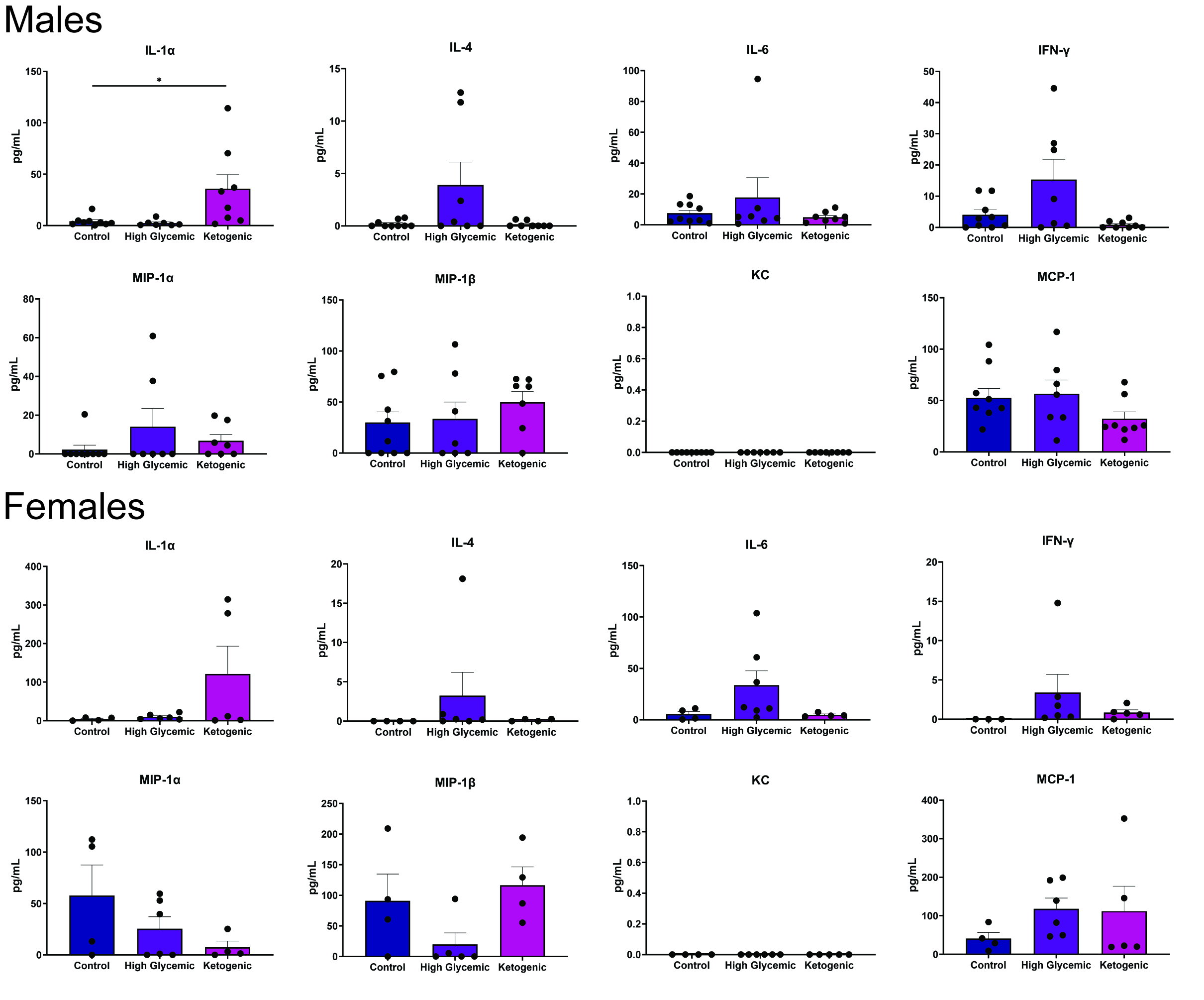

Supplement: Supplementary file 4 — Additional file 4. Inflammatory cytokine/chemokine levels in serum collected from 26-week-old mice. Mice were started on a control, high glycemic index, or ketogenic diet at 6 weeks of age and serum was isolated at 26 weeks. Cytokine/chemokine were measured by CBA in male and female mice. All data are shown as mean ± SEM and n = 3–10 per group. Statistical differences were determined using one-way ANOVA followed by a Dunnett’s test, comparing the HGD or KD to their sex-matched CD. *p < 0.05. [file 12974_2024_3140_MOESM4_ESM.tif]

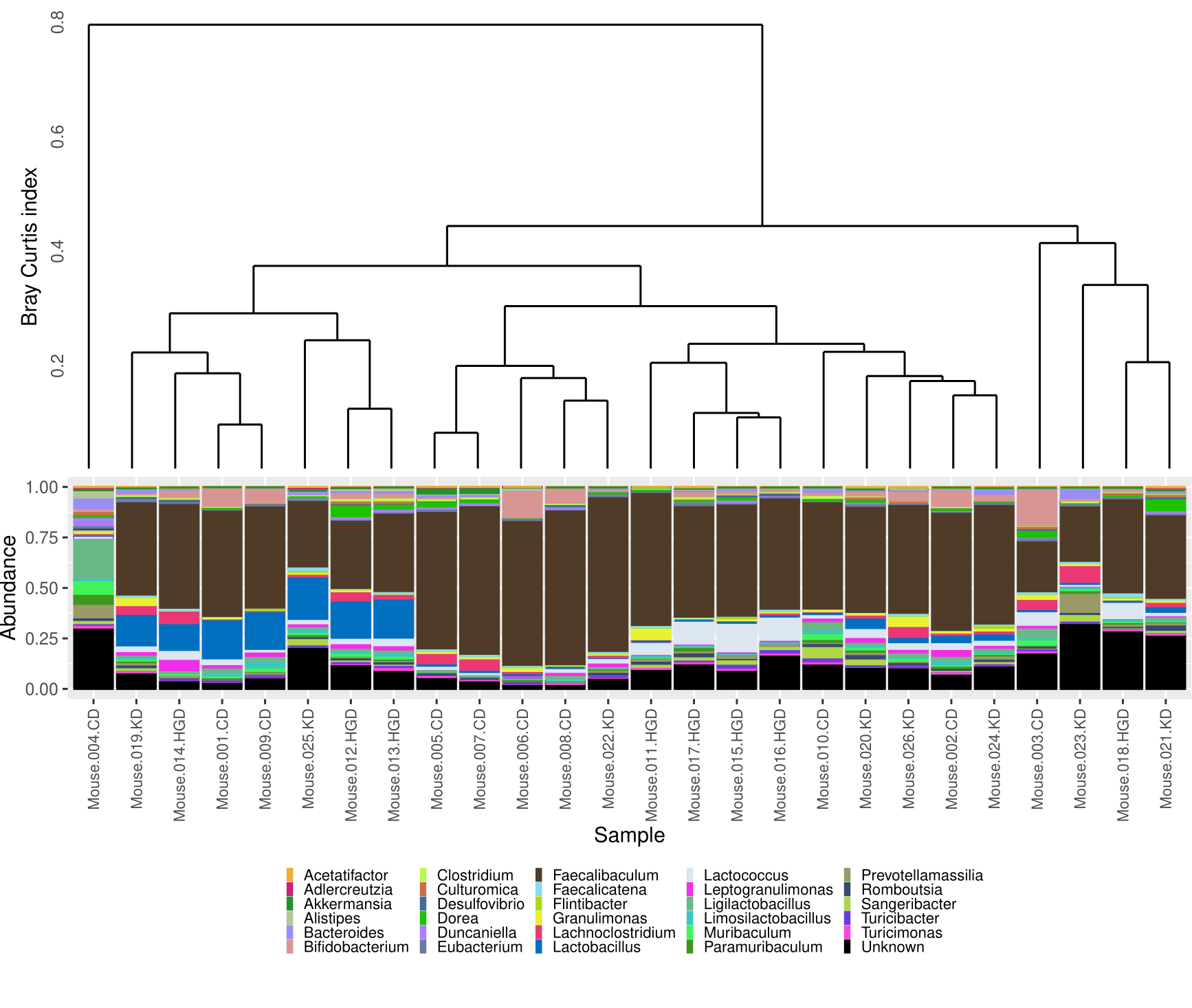

Supplement: Supplementary file 5 — Additional file 5. Relative abundance clustered by the Bray-Curtis Dissimilarity Index for all samples. DNA was extracted from 26-week-old male mice and 16S ribosomal RNA gene amplicon sequencing performed. The Bray-Curtis Dissimilarity Index identified 1 control diet sample (sample 4) that was substantially different and therefore excluded from further analysis. N = 8–10 per group. [file 12974_2024_3140_MOESM5_ESM.tif]

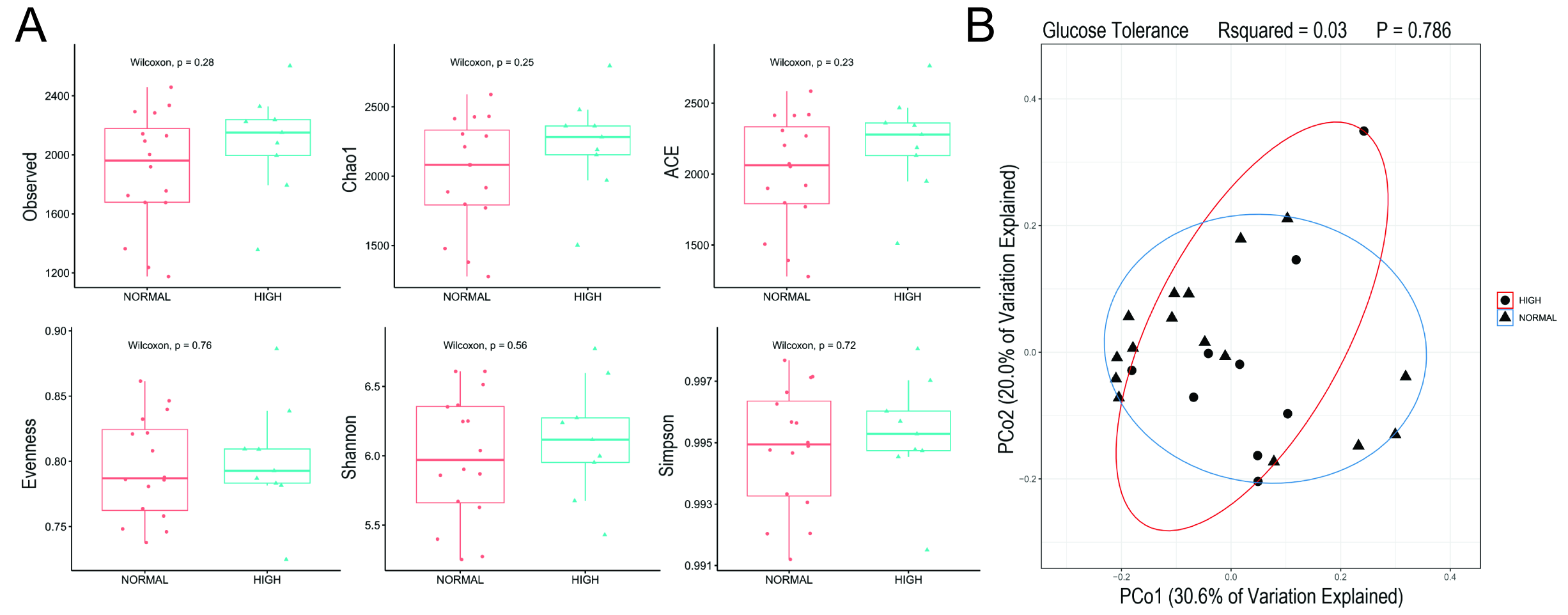

Supplement: Supplementary file 6 — Additional file 6. Microbiome composition does not fully explain glucose intolerance phenotype. DNA was extracted from 26-week-old male mice and 16S ribosomal RNA gene amplicon sequencing performed. Raw measurements of area under the curve for the glucose tolerance test were binned into either normal or high and used as metadata in analyses. (A) Alpha diversity analysis was performed by calculating richness (ace, chao1, and observed richness), evenness (Pielou) and overall diversity (Shannon and Simpson). (B) Beta diversity was assessed by PCoA using the Bray-Curtis Dissimilarity Index at the ASV level and the resultant values tested for significance with the experimental covariates using the PERMANOVA test. N = 8–10 per group. Statistical comparisons of bacterial relative abundance were conducted using the Phyloseq package and involved pairwise Chi-Squared Tests, and results were filtered based on a significance threshold of Benjamini-Hochberg adjusted p-value < 0.01. [file 12974_2024_3140_MOESM6_ESM.tif]

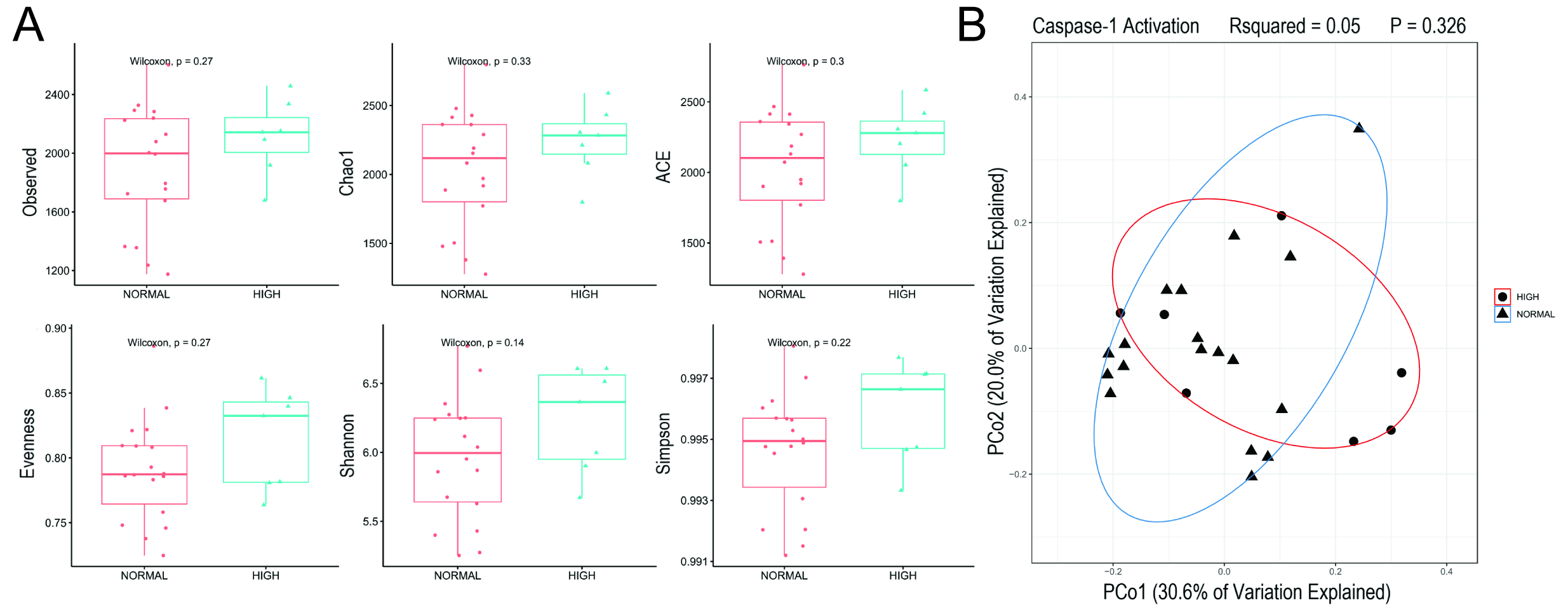

Supplement: Supplementary file 7 — Additional file 7. Microbiome composition does not fully explain caspase-1 activation in the CNS. DNA was extracted from 26-week-old male mice and 16S ribosomal RNA gene amplicon sequencing performed. Raw measurements of total flux for caspase-1 activation were binned into either normal or high and used as metadata in analyses. (A) Alpha diversity analysis was performed by calculating richness (ace, chao1, and observed richness), diversity (Shannon and Simpson), and evenness (Pielou). (B) Beta diversity was assessed by PCoA using Bray-Curtis dissimilarities at the ASV level and dissimilarity indices were tested for significance with the experimental covariates using the PERMANOVA test. N = 8–10 per group. Statistical comparisons of bacterial abundance were conducted using the Phyloseq package and involved pairwise Chi-Squared Tests, and results were filtered based on a significance threshold of Benjamini-Hochberg adjusted p-value < 0.01. [file 12974_2024_3140_MOESM7_ESM.tif]
